# The Content of Phenolic Compounds in Leaf Tissues of *Aesculus glabra* and *Aesculus parviflora* Walt.

**DOI:** 10.3390/molecules20022176

**Published:** 2015-01-28

**Authors:** Jan Oszmiański, Joanna Kolniak-Ostek, Agata Biernat

**Affiliations:** 1 Department of Fruit, Vegetable and Grain Technology, Wroclaw University of Environmental and Life Sciences, 37/41 Chełmońskiego St., Wroclaw 51-630, Poland; E-Mail: jan.oszmianski@up.wroc.pl; 2 Department of Biology and Pharmaceutical Botany with Garden of Medicinal Plants, University of Medicine in Wrocław, Kochanowskiego 12, Wrocław 51-601, Poland; E-Mail: agata.biernat@umed.wroc.pl

**Keywords:** *Aesculus*, polyphenols, polymeric procyanidins, leaves, *Cameraria ohridella*, LC-MS QTof

## Abstract

In plants, flavonoids play an important role in biological processes. They are involved in UV-scavenging, fertility and disease resistance. Therefore, in this study, we attempted to quantify and characterize phenolic compounds in *Aesculus parviflora* Walt. leaves and *Aesculus glabra* leaves partly suffering from attack by a leaf mining insect (*C. ohridella*). A total of 28 phenolic compounds belonging to the hydroxycinnamic acid, flavan-3-ols and flavonol groups were identified and quantified in *Aesculus parviflora* and *A*. *glabra* leaf extracts. Significantly decreased concentrations of some phenolic compounds, especially of flavan-3-ols, were observed in infected leaves compared to the non-infected ones. Additionally, a higher content of polymeric procyanidins in leaves of *Aesculus parviflora* than in *Aesculus glabra* may explain their greater resistance to *C. ohridella* insects.

## 1. Introduction

Flavonoids represent a large family of low molecular weight polyphenolic secondary metabolites that are widespread throughout the plant kingdom [1]. In plants, flavonoids play an important role in biological processes. Beside their function as pigments in flowers and fruits, to attract pollinators and seed dispersers, flavonoids are involved in UV-scavenging, fertility and disease resistance [2]. Contrary to animals, plants cannot synthesize antibodies for defense, but can produce numerous phenolic substances, which are a part of the complex immune system. Polyphenols, which can be acquired in tissues under stress, are key components of active and potent defense mechanisms against pests and pathogens [3]. The involvement of phenols in plant disease resistance is based, to a large extent, on their cytotoxicity, which is associated with their oxidation products [4]. It has been proposed that the first stage of the defense mechanism of plants involves a rapid accumulation of phenols at the infection site, which function to slow down the growth of the pathogens. Polyphenols play a vital role in the growth and propagation of plants and protect plant tissues from damage. A number of phenols are regarded as pre-infection inhibitors, providing plants with a certain degree of basic resistance against pathogenic microorganisms [5]. The secondary compounds in plants are strongly involved in the interaction between the pathogen and the plant. In many works [6,7,8,9], there is a strong correlation between high phenolic concentrations and resistance against herbivores, including insect pests.

*Aesculus* L. is a genus of the family Hippocastanaceae containing 12 species of deciduous trees and shrubs in the Northern Hemisphere, primarily in eastern Asia and eastern North America, with one species native to Europe and two to western North America [10,11]. To date, over a hundred species or varieties in *Aesculus* have been described because of the great morphological variations due to natural pollination among the species.

*Aesculus glabra*, also known as American buckeye, Ohio buckeye, fetid buckeye and stinking buckeye, derives its unflattering common names from the disagreeable odor that emanates when the leaves are crushed. The tree is an attractive ornamental, but it has limited commercial use, because of the soft, light wood and presence of narcotic glucoside in bark and seeds. This species generally does not grow taller than 9.1 m and seldom exceeds 21.3 m [12]. It has been planted in Europe and the eastern United States (in eastern Massachusetts, Minnesota and western Kansas) [13].

*Aesculus parviflora*, also known as bottlebrush buckeye, is also called “dwarf horse chestnut” in recognition of its resemblance to its more famous relative, *A. hippocastanum*. It is a deciduous suckering shrub growing to 3–5 m tall, native to open woodlands of the southeastern United States. It is grown as an ornamental plant in gardens [14].

Over the past two decades, this particular tree species has suffered heavily from attacks by a leaf mining insect, known as *Cameraria ohridella* Deschka & Dimić, the horse chestnut leaf-miner [15]. Damage is caused by the larvae, which mine the leaves of the host plant between the epidermis layers. Two to four generations can occur, depending on climatic conditions [16,17,18], causing premature browning and leaf fall. Eventually, leaves die and fall prematurely. If new leaves develop, they can be re-infected [19,20]. The appearance and mass occurrence of the horse chestnut leaf miner (*C. ohridella*) has had a negative effect on decorativeness of the attacked trees. This weakened condition of the infested trees makes them more susceptible to other enemies and diseases, which would not be harmful in normal circumstances. *Cameraria ohridella* primarily attacks and develops on *Aesculus* spp., but the moth has also been found to develop to adults on the closely-related genus, *Acer*, in particular on the European *A. pseudoplatanus* L. and *A. platanoides* L. [21,22]. Freise *et al.* [21] investigated the physiological host range of *C. ohridella* by testing the moth on 36 taxa of species, cultivars and hybrids of *Aesculus* spp. Considerable variation exists in the susceptibility to *C. ohridella* between *A. hippocastanum* cultivars, e.g., cv. *Pyramidalis* is reported to be more susceptible than cv. *Memmingeri* [23].

The presence of phenolic compounds in *A. hippocastanum* had been reported in the literature [24]. The antioxidant activity of the seeds was attributed to the presence of flavonoids, in particular glycoside and acylated forms of quercetin and kaempferol [25,26]. One of the strategies of physiological defense against phytophagous insects is the production of larger amounts of tannins. Tannins are derivatives of phenolic compounds with a bitter taste and toxic properties that can discourage or deter animals from feeding. The presence of catechin tannins has been found in horse chestnut leaves in our previous research [27]. A higher content of polymeric procyanidins in the leaves of red-horse chestnut than in the leaves of white-horse chestnut may explain their greater resistance to *C. ohridella* insects and can be attributable to the fact that the larvae do avoid the tannin-containing tissues, e.g., the epidermis.

To understand the role of polyphenolic compounds, especially polymeric procyanidins in defense mechanisms against leaf miner larvae, *Aesculus* leaves were investigated. In order to find out whether the phenolic compounds are different in infested leaves, we investigated two *Aesculus* species, with diverse resistance to mining insects. Therefore, the aim of this study was to identify and quantify the main phenolic compounds in *Aesculus parviflora* Walt. leaves, which were resistant to larvae attack, and *Aesculus glabra* leaves, partly infested by a leaf mining insect (*C. ohridella*).

## 2. Results and Discussion

### 2.1. Identification of Phenolics in *Aesculus* Leaves

**Figure 1:**
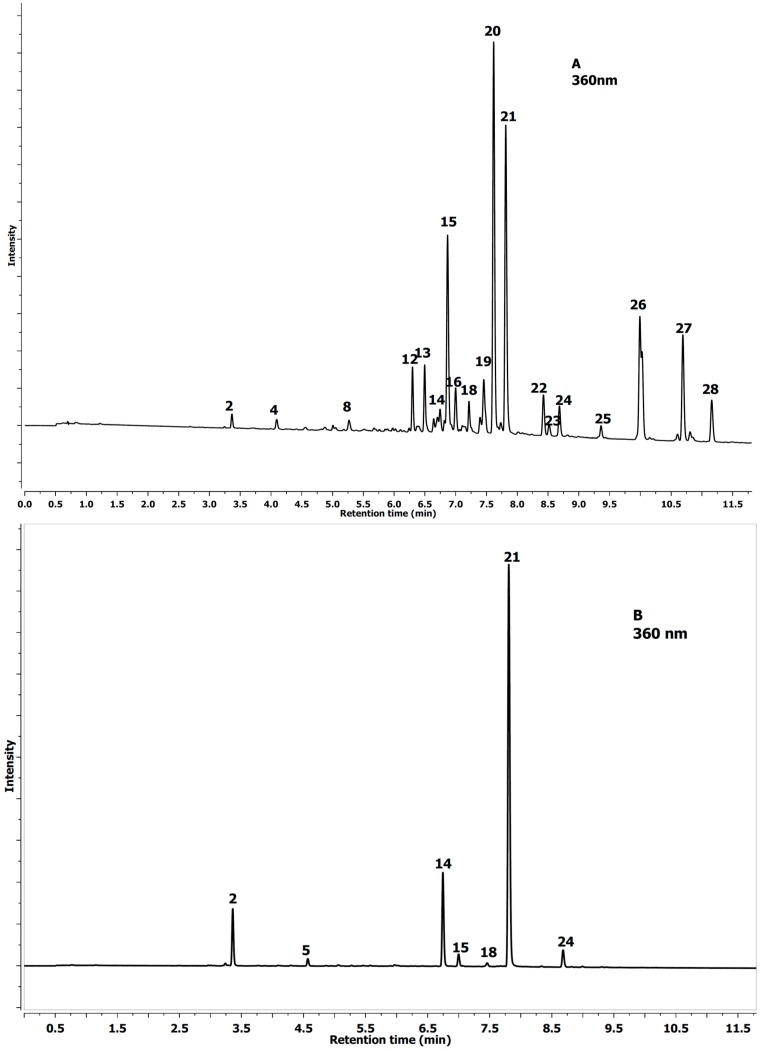
UPLC chromatogram profile of *Aesculus glabra* (**A**) and *Aesculus parviflora* Walt.; (**B**) chestnut leaf extracts at 360 nm (phenolic acid and quercetin derivative). See Table 1 for the peak labels.

**Figure 2:**
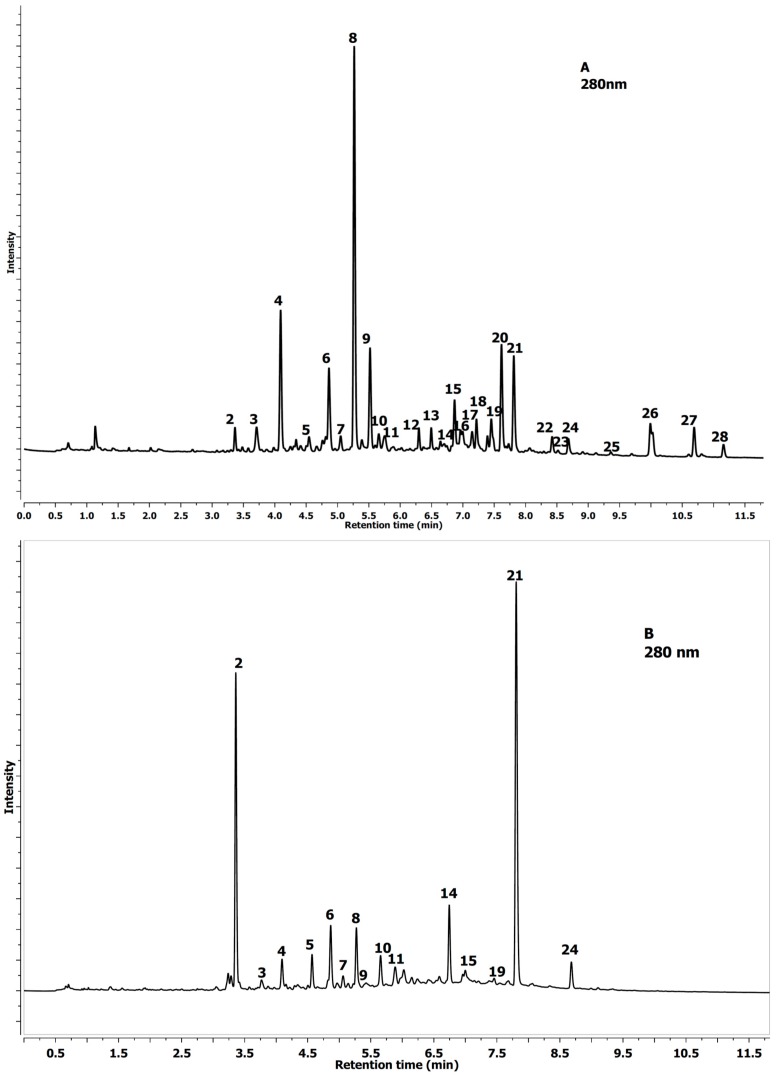
UPLC chromatogram profile *Aesculus glabra* (**A**); and *Aesculus parviflora* Walt.; (**B**) leaf extracts at 280 nm (flavonols).

The extracts from *Aesculus parviflora* leaves and *Aesculus glabra* leaves partly suffering from attack by a leaf mining insect (*C. ohridella*) were analyzed by an LC-ESI-MS/MS system. Results of qualitative analysis obtained by LC-PDA-MS/MS methods and quantitative analysis obtained by UPLC-PDA are summarized in Table 1 and Table 2 and Figure 1 and Figure 2. A total of 28 phenolic compounds found in *Aesculus* leaf extracts were identified and are presented.

Four hydroxycinnamates were detected, and two of them, neochlorogenic (peak **2**) and chlorogenic (peak **5**) acid, were identified by comparison with authentic standards (Table 1). Those compounds had [M−H]^−^ at *m*/*z* 353 and fragmentation at *m*/*z* 191, corresponding to quinic acid and λmax 324 nm. Compound **1** has [M−H]^−^ at m/z 315 and fragmentation at *m*/*z* 153, corresponding to the loss of the hexose residue (162 Da) and formation of protocatechuic acid aglycone. Peak **4** has *m/z* at 337, λmax 309 nm and fragmentation at 163, corresponding to the coumarate ion. This compound was identified as 3-*O*-*p*-coumaroylquinic acid.

**Table 1:** The characterization of phenolic compounds of the *Aesculus* leaves using their spectral characteristic in UPLC-DAD (retention time, λ_max_) and negative ions in UPLC-ESI-MS.

No Peak	Tentative Identification	Rt (min)	λ_max_ (nm)	[MS]^−^ *m*/*z*	[MS−MS]^−^ *m*/*z*
**1**	Protocatechuic-acid-4-glucoside	2.69	292	315.0719	153.0180
**2**	Neochlorogenic acid ^a^	3.37	324	353.0879	191.0552
**3**	Procyanidin tetramer A-type	3.71	280	1151.2501	289.0718
**4**	3-*O*-*p*-Coumaroylquinic acid	4.09	309	337.0928	163.0394
**5**	Chlorogenic acid ^a^	4.55	324	353.0888	191.0554
**6**	Procyanidin B1 ^a^	4.86	280	577.1337	289.0708
**7**	Procyanidin tetramer B-type	5.05	280	1153.2748	289.0713
**8**	(−)-Epicatechin ^a^	5.27	280	289.0719	245.0818
**9**	A-type PA-trimer	5.52	277	863.1826	289.0714
**10**	B-type PA-trimer	5.66	280	865.2001	289.0726
**11**	Procyanidin tetramer A-type	5.76	280	1151.2525	289.0726
**12**	Quercetin-glucoside-rhamnoside-rhamnoside	6.32	350	755.2052	301.0413
**13**	Quercetin-*O*-deoxyhexose-*O*-deoxyhexoside	6.50	350	593.1505	301.0708
**14**	Quercetin-3-*O*-rutinoside ^a^	6.74	350	609.1461	301.0393
**15**	Quercetin-3-*O*-galacoside ^a^	6.87	355	463.0873	301.0352
**16**	Quercetin-3-*O*-glucoside	7.00	355	463.0878	301.0355
**17**	Procyanidin A-type dimer	7.14	280	575.1195	289.0717
**18**	Kaempferol-3-*O*-rutinoside	7.20	350	593.1506	285.0409
**19**	Quercetin-deoxyhexoside-hexoside	7.46	350	609.1449	301.0355
**20**	Quercetin-3-*O*-arabinoside	7.62	350	433.0773	301.0351
**21**	Quercetin-3-*O*-rhamnoside ^a^	7.83	350	447.0926	301.0353
**22**	Keampferol-3-*O*-arabinoside	8.43	350	417.0826	285.0401
**23**	Kaempferol glucuronide	8.53	350	461.0716	285.0386
**24**	Keampferol-3-*O*-rhamnoside	8.71	350	431.0978	285.0399
**25**	Isorhamnetin-3-*O*-rutinoside	9.39	350	632.1630	315.0505
**26**	Isorhamnetin-3-*O*-glucoside	9.99	350	477.1027	315.0497
**27**	Isorhamnetin-3-*O*-pentoside	10.69	350	447.0924	315.0504
**28**	Isorhamnetin-3-*O*-rhamnoside	11.16	350	461.1084	315.0493

Note: ^a^ Identification confirmed by commercial standards.

Eight flavan-3-ols were detected in *Aesculus* leaf extracts: (−)-epicatechin, four A-type procyanidins (one dimer, one trimer and two tetramers) and three B-type procyanidins (procyanidin B_1_, one trimer and one tetramer) (Table 1). Compounds **6** and **8** were identified as procyanidin B_1_ and (−)-epicatechin, respectively. Their retention times at 4.86 min and 5.27 min and λmax at 280 nm were compared with standards. The B-type procyanidin trimer (Peak **10**) and tetramer (Peak **7**) were identified in *Aesculus* leaf extracts, as well. These compounds had pseudomolecular ions at *m*/*z* 865 and *m*/*z* 1153, respectively, and fragmentation ions at *m*/*z* 289, corresponding to the monomer of catechins. Compound **17** has a [M−H]^−^ at *m*/*z* 575, a fragmentation ion at *m*/*z* 289 and λmax at 280 nm. This compound was identified as an A-type procyanidin dimer. The A-type procyanidins trimer (Peak **9**) and tetramers (Peaks **3** and **11**) showed UV-spectrum λmax = 280 nm and ions at *m*/*z* 863 and 1151, respectively. (−)-Epicatechin and its A-type proanthocyanidins, have been reported in *Aesculus* *hippocastanum* and *carea* leaves in our previous research [27].

**Table 2:** The content (mg/g dm) of phenolic compounds of the extracts of *Aesculus glabra* and *Aesculus parviflora* Walt. leaves.

Compounds ^£^	*Aesculus glabra*	*Aesculus parviflora* Walt.
Protocatechuic-acid-4-glucoside	0.42 ± 0.01 s *	0.00 ± 0.00 v
Neochlorogenic acid	1.53 ± 0.06 m	9.62 ± 0.12 e
Procyanidin tetramer A-type	3.08 ± 0.08 i	0.50 ± 0.00 r
3-*O*-*p*-Coumaroylquinic acid	3.70 ± 0.03 i	0.43 ± 0.00 s
Chlorogenic acid	1.60 ± 0.01 l	1.47 ± 0.01 m
Procyanidin B1	11.73 ± 0.15 d	4.47 ± 0.12 h
Procyanidin tetramer B-type	2.11 ± 0.00 j	0.80 ± 0.00 p
(−)-Epicatechin	51.54 ± 0.22 b	3.84 ± 0.13 i
A-type PA-trimer	8.90 ± 0.10 e	0.00 ± 0.00 v
B-type PA-trimer	1.88 ± 0.01 k	2.07 ± 0.01 j
Procyanidin tetramer A-type	1.91 ± 0.02 j	0.08 ± 0.01 u
Q-glu-rha-rha	0.73 ± 0.00 q	0.00 ± 0.00 v
Quercetin-*O*-deoxyhexose-*O*-deoxyhexoside	0.78 ± 0.00 pq	0.00 ± 0.00 v
Quercetin-3-*O*-rutinoside	0.57 ± 0.00 r	4.07 ± 0.12 h
Quercetin-3-*O*-galacoside	4.05 ± 0.11 h	0.42 ± 0.08 s
Quercetin-3-*O*-glucoside	1.31 ± 0.01 n	0.00 ± 0.00 v
Procyanidin A-type dimer	1.64 ± 0.10 l	0.00 ± 0.00 v
Kaempferol-3-*O*-rutinoside	0.85 ± 0.01 p	0.24 ± 0.09 t
Quercetin-deoxyhexoside-hexoside	1.92 ± 0.13 jk	0.00 ± 0.00 v
Quercetin-3-*O*-arabinoside	9.14± 0.21 e	0.00 ± 0.00 v
Quercetin-3-*O*-rhamnoside	7.31 ± 0.10 f	15.40 ± 0.15 d
Keampferol-3-*O*-arabinoside	1.54 ± 0.03 m	0.00 ± 0.00 v
Kaempferol glucuronide	0.16 ± 0.01 t	0.00 ± 0.00 v
Keampferol-3-*O*-rhamnoside	1.19 ± 0.00 o	1.06 ± 0.07 o
Isorhamnetin-3-*O*-rutinoside	0.38 ± 0.00 s	0.00 ± 0.00 v
Isorhamnetin-3-*O*-glucoside	5.52 ± 0.10 g	0.00 ± 0.00 v
Isorhamnetin-3-*O*-pentoside	2.95 ± 0.02 ij	0.00 ± 0.00 v
Isorhamnetin-3-*O*-rhamnoside	1.14 ± 0.03 o	0.00 ± 0.00 v
Procyanidin polymers	37.04 ± 0.20 c	81.87 ± 0.25 a
Total	166.62 A	126.34 B

Notes: * Values are the means ± standard deviation, *n* = 3; mean values with different letters are significantly different at *p* < 0.05; ^£^ amounts of hydroxycinnamic acids, flavan-3-ols and flavonols were converted into chlorogenic acid (hydroxycinnamic acids), (–)-epicatechin (flavan-3-ols), quercetin-3-*O*-galactoside (quercetin derivatives), kaempferol-3-*O*-galactoside (kaempferol derivatives) and isorhamnetin-3-*O*-galactoside (isorhamnetin derivatives).

Sixteen flavonols were detected in the *Aesculus* leaf extracts: eight quercetin derivatives (Peaks **12**–**16** and **19**–**21**), four kaempferol derivatives (Peaks **18** and **22**–**24**) and four isorhamnetin derivatives (Peaks **25**–**28**). Quercetin derivatives were represented by: quercetin-3-*O*-rhamnoglucoside (rutinoside), 3-*O*-rutinoside-rhamnoside, *O*-deoxyhexoside-*O*-deoxyhexoside, *O*-deoxyhexoside-*O*-hexoside, 3-*O*-galactoside, 3-*O*-glucoside, 3-*O*-arabinoside and 3-*O-*rhamonside. All compounds had a typical quercetin ion fragment at *m*/*z* 301. These compounds were compared with retention times, UV-Vis absorption spectra and mass fragmentation of quercetin-3-*O*-galactoside, 3-*O*-rutinoside and quercetin-3-*O*-rhamnoside standards, for the more detailed identification of the sugar units of quercetin glycosides. Four derivatives of kaempferol, 3-*O*-rutinoside, 3-*O*-arabinoside, glucuronide and 3-*O*-rhamnoside, were detected. These peaks had the typical kaempferol ion fragment at *m*/*z* 285 and [M−H]^−^ at *m*/*z* 593, 417, 461 and 431, respectively. Four isorhamnetin derivatives were represented by: isorhamnetin-3-*O*-rutinoside, 3-*O*-glucoside, 3-*O*-penoside and 3-*O*-rhamnoside. These compounds had a [M−H]^−^ at *m*/*z* 632, 477, 447 and 461, respectively, and the typical isorhamnetin ion fragment at *m*/*z* 315.

Derivatives of quercetin and kaempferol have been reported in *Aesculus* *hippocastanum* and *carea* leaves in our previous research [27]. In addition, these flavonols and their glycosides have been detected and characterized in the seeds of *A. hippocastanum* and *A. chinensis* [25,26,28]. Derivatives of isorhamnetin were also isolated and identified from the leaves of *A. pa*v*ia*, grown in Italy [29].

### 2.2. Quantification of Phenolics in *Aesculus* Leaves

The content of phenolics in *Aesculus parviflora* leaves and *Aesculus glabra* leaves partly suffering from attack by a leaf mining insect varied significantly, as shown in Table 2. The total content of phenolics in *A. glabra* was 166.62 mg/g dm. At the same time, in the non-infected *A. parviflora* leaves, the concentration of polyphenolic compounds was significantly lower and reached 126.34 mg/g dm. Leaves of *A. glabra* were characterized by a higher content of flavonols and procyanidin monomers and oligomers than *A. parviflora* leaves. In contrast, *A. parviflora*, which was more resistant to mining insects, contained more polymeric procyanidins than *A. glabra*, *i.e.*, 81.87 mg and 37.04 mg/g dm, respectively. A higher concentration of polymeric tannins in *A. parviflora* leaves than on *A. glabra* leaves may explain their greater resistance to *C. ohridella* insects. Similar results were obtained in our previous research [27]. In leaves of white and red chestnut horse leaves that suffered from attack by a leaf mining insect, the concentration of polymeric procyanidins was significantly lower than in non-infected ones. 

The difference between *A. glabra* and *A. parviflora* leaves is depicted in Figure 1 and Figure 2, which show a much greater differentiation of the peaks.

Plant phenolics are secondary metabolites involved in the defense mechanisms of plants against fungal pathogens and insect herbivores. Niederleitner *et al.* [30] found that infected sour cherry leaves contained higher concentrations of (+)-catechin, (−)-epicatechin and procyanidins B_2_, B_5_ and C_1_ than non-infected ones. A transient accumulation of some compounds, especially flavan-3-ols, was also detected after pear leaf wounding, thus confirming the role of phenolic compounds in resistance to injury and stress [31]. Schultz [32] reported that the chemical composition of *Betula lutea* and *Acer saccharum* leaves affected the resistance to pest damage. The leaves, which contain less tannins, were damaged more by pests, in comparison to those with a high tannin concentration.

Numerous scientific studies show that polyphenols, and specifically tannins, are plant defense compounds against insect herbivores [33,34,35]. Tannins present the capacity to interact and precipitate a wide array of molecules, including proteins [36]. This capacity was the basis for suggesting that tannins affected insect herbivores by inactivating insect enzymes, as well as dietary proteins [37,38]. Furthermore, some flavonols may have an affect against herbivores. Isman and Duffey [39] in their studies show that quercetin-3-*O*-rutinoside deterred the *Heliothis zea* larvae from feeding.

## 3. Experimental Section

### 3.1. Reagent and Standards

Formic acid and methanol were purchased from Sigma-Aldrich (Steinheim, Germany). Acetonitrile was purchased from Merck (Darmstadt, Germany). Qercetin-3-*O*-galactoside, quercetin-3-*O*-rhamnoside, quercetin-3-*O*-rutinoside, kaempferol-3-*O*-glucoside, (–)-epicatechin and procyanidin A_2_ and B_2_ were purchased from Extrasynthese (Lyon, France). Neochlorogenic acid and caffeic acid were purchased from TRANS MIT GmbH (Giessen, Holland).

### 3.2. Plant Material

The leaves of *Aesculus glabra* and *Aesculus parviflora* Walter were collected from the Garden of Medicinal Plants at the Medical University in Wroclaw, Poland. Leaves of *A. glabra*, which were infected with *C. ohridella*, and leaves of *A. parviflora*, which were more resistant to leaf miner larvae attack, were picked up on the same day in early July, 2014. In the course of the measurements, 3 replications (10 randomly-chosen leaves) from 3 trees, that is 30 replications, were established. The condition of infected leaves was evaluated based on their appearance. After harvest, the leaves were cut and directly frozen in liquid nitrogen and freeze-dried (24 h; Christ Alpha 1-4 LSC; Martin Christ GmbH, Osterode am Harz, Germany). Homogeneous powders were obtained by crushing the dried tissues using a closed laboratory mill to avoid hydration (IKA 11A; Staufen, Germany). Powders were kept in a refrigerator (−80 °C) until extract preparation.

### 3.3. Extraction Procedure

The powder samples (1 g) were extracted with 25 mL of methanol acidified with 1%.acetic acid. The extraction was performed twice by incubation for 20 min under sonication and with occasional shaking. Next, the slurry was centrifuged at 19,000× *g* for 10 min, and the supernatant was filtered through a 0.25-µm membrane and used for analysis. The identification and content of polyphenols in individual extracts was determined by means of the liquid chromatography (UPLC-PDA and LC/MS) method.

### 3.4. Identification of Polyphenols by the Liquid Chromatography-Mass Spectrometry (LC-MS) Method

The identification of the polyphenols of *Aesculus* leaf extracts was carried out using an ACQUITY Ultra Performance LC™ system with a mass detector G2 QTof Micro mass spectrometer equipped with an electrospray ionization (ESI) source operating in negative mode (UPLC™; Waters Corporation, Milford, MA, USA). The separation of individual polyphenols was carried out using a UPLC BEH C18 column (1.7 μm, 2.1 × 100 mm, Waters Corporation) at 30 °C. The elution solvents were aqueous 0.1% formic acid (A) and 100% acetonitrile (B). Samples (10 μL) were eluted according to the linear gradient described by Oszmiański *et al.* [40]. Analysis was carried out using full scan, data-dependent MS scanning from *m*/*z* 100 to 1500. The mass tolerance was 0.001 Dalton, and the resolution was 5.000. Leucine enkephalin was used as the internal reference compound during ESI-MS accurate mass experiments and was permanently introduced via the LockSpray channel using a Hamilton pump. The lock mass correction was +/– 1.000 for the mass window. All TOF-MS chromatograms are displayed as base peak intensity (BPI) chromatograms and scaled to 12,400 counts per second (cps) (= 100%). The effluent was led directly to an electrospray source with a source block temperature of 130 °C, a desolvation temperature of 350 °C, a capillary voltage of 2.5 kV and a cone voltage of 30 V. Nitrogen was used as the desolvation gas with a flow rate of 300 L·h^−1^.

The characterization of the single components was carried out via their retention time and the accurate molecular masses. Each compound was optimized to its estimated molecular mass [M−H]^−^ in negative mode before and after fragmentation. The data obtained from LC/MS were subsequently entered into the MassLynx 4.0 ChromaLynx™ Application Manager software. Based on these data, the software is able to scan different samples for the characterized substances.

The runs were monitored at the following wavelengths: (–)-epicatechin and procyanidin at 280 nm, hydroxycinnamates at 320 nm and flavonol glycosides at 360 nm. Retention times (R_t_) and spectra were compared with those of pure standards. Calibration curves at concentrations ranging from 0.05 to 5 mg/mL (*r*^2^ ≤ 0.9998) were made from (−)-epicatechin, procyanidin A_2_, procyanidin B_2_, neochlorogenic acid, caffeic acid, quercetin-3-*O*-galactoside, quercetin-3-*O*-rutinoside and kaempferol-3-*O*-glucoside as the standards. All incubations were done in triplicate. The results was expressed as milligrams per g of dry matter (dm).

### 3.5. Analysis of Proanthocyanidins by the Phloroglucinolysis Method

Direct phloroglucinolysis of freeze-dried *Aesculus* leaves was performed as described previously by Kolniak-Ostek *et al.* [41]. Portions (0.05 g) of powder were precisely measured into 2-mL Eppendorf vials, then a methanolic solution (0.8 mL) of phloroglucinol (75 g/L) and ascorbic acid (15 g/L) was added. After the addition of methanolic HCl (0.4 mL, 0.3 mol/L), the vials were closed and incubated for 30 min at 50 °C with continuous vortexing using a thermo shaker (TS-100; BIOSAN, Riga, Lithuania). The reaction was stopped by placing the vials in an ice bath by drawing 0.5 mL of the reaction medium and diluting with 0.5 mL of 0.2 mol/L sodium acetate buffer. Next, the vials were cooled in ice water and centrifuged immediately at 20,000× *g* for 10 min at 4 °C. The analytical column was kept at 15 °C by the column oven, whereas the samples were kept at 4 °C. The analysis of polymeric procyanidin compounds was carried out on an Acquity UPLC system (Waters Corp.) consisting of a binary solvent manager and fluorescence detector (FL). Empower 3 software was used for chromatographic data collection and integration of chromatograms. A partial loop injection mode with a needle overfill was set up, enabling 5-μL injection volumes when a 10-μL injection loop was used. Acetonitrile (100%) was used as a strong wash solvent and acetonitrile-water (10%) as a weak wash solvent. The analytical column BEH Shield C18 (2.1 mm × 50 mm; 1.7 μm) was kept at 15 °C by a column oven, whereas the samples were kept at 4 °C. The flow rate was 0.45 mL/min. The mobile phase was composed of Solvent A (2.5% acetic acid) and Solvent B (acetonitrile). Elution was as follows: 0–0.6 min, isocratic 2% B; 0.6–2.17 min, linear gradient from 2% to 3% B; 2.17–3.22 min, linear gradient from 3% to 10% B; 3.22–5.00 min, linear gradient from 10% to 15% B; 5.00–6.00 min, column washing; and reconditioning for 1.50 min. The fluorescence detection was recorded at an excitation wavelength of 278 nm and an emission wavelength of 360 nm. The calibration curves, which were based on peak area, were established using (+)-catechin, (−)-epicatechin and procyanidin B_1_ after phloroglucinol reaction as (+)-catechin- and (−)-epicatechin-phloroglucinol adduct standards. The average degree of polymerization was calculated as the molar ratio of all of the flavan-3-ol units (phloroglucinol adducts + terminal units) to (−)-epicatechin and (+)-catechin, which correspond to terminal units. All incubations were done in triplicate. The results was expressed as milligrams per g dm.

### 3.6. Statistical Analysis

Results are presented as the mean ± standard deviation of three technical replications. All statistical analyses were performed with Statistica version 10.0 (StatSoft, Tulsa, OK, USA). One-way analysis of variance (ANOVA) by Duncan’s test was used to compare the mean values. Differences were considered to be significant at *p* < 0.05.

## 4. Conclusions

In plants, flavonoids play an important role in biological processes. Normally, they are secondary metabolites involved in the defense mechanisms of plants against pathogens. In our research, we compared infected and non-infected leaves of *Aesculus glabra* and *Aesculus parviflora*. We observed a significantly decreased concentration of some phenolic compounds in leaves after infection, especially flavan-3-ols. Additionally, a higher content of polymeric procyanidins in leaves of *Aesculus parviflora* than in *Aesculus glabra* may explain their greater resistance to *C. ohridella* insects. Based on the data obtained, it may be speculated that the activity of some polyphenolic compounds plays a vital role in resistance to injury and stress.
